# Drone-Based Harvest Data Prediction Can Reduce On-Farm Food Loss and Improve Farmer Income

**DOI:** 10.34133/plantphenomics.0086

**Published:** 2023-09-07

**Authors:** Haozhou Wang, Tang Li, Erika Nishida, Yoichiro Kato, Yuya Fukano, Wei Guo

**Affiliations:** ^1^ Graduate School of Agricultural and Life Sciences, The University of Tokyo, Tokyo, Japan.; ^2^ Graduate School of Horticulture, Chiba University, Chiba, Japan.

## Abstract

On-farm food loss (i.e., grade-out vegetables) is a difficult challenge in sustainable agricultural systems. The simplest method to reduce the number of grade-out vegetables is to monitor and predict the size of all individuals in the vegetable field and determine the optimal harvest date with the smallest grade-out number and highest profit, which is not cost-effective by conventional methods. Here, we developed a full pipeline to accurately estimate and predict every broccoli head size (*n* > 3,000) automatically and nondestructively using drone remote sensing and image analysis. The individual sizes were fed to the temperature-based growth model and predicted the optimal harvesting date. Two years of field experiments revealed that our pipeline successfully estimated and predicted the head size of all broccolis with high accuracy. We also found that a deviation of only 1 to 2 days from the optimal date can considerably increase grade-out and reduce farmer's profits. This is an unequivocal demonstration of the utility of these approaches to economic crop optimization and minimization of food losses.

## Graphical abstract

**Figure:**
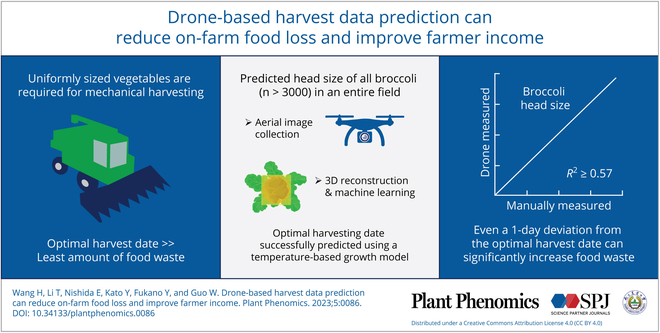

## Introduction

Waste caused by nonstandard vegetables is an unavoidable component of food loss in modern society [1,2]. Vegetables that do not meet the cosmetic standards (e.g., size, shape, and aesthetics) cannot be easily sold and are not harvested [3]. Porter et al. [4] estimated that over one-third of the total agricultural production (e.g., 51,500 kilotons annually in the European Economic Area) is lost. For example, broccoli (*Brassica oleracea* L.) head is an important component of the global vegetable market with a high percentage of on-farm waste. Its nonedible parts (leaves, stems) account for over 75% of the above-ground biomass (Table 1 of [5]). For the remaining marketable parts, the variable bud growth rate results in large variations in head size under complex field conditions. Therefore, a certain amount of nonstandard harvested head is wasted from one-time mechanical harvesting. Although the conventional method of selective harvesting by hand during the growing season could minimize such waste, the labor cost (107 person-hours per hectare) eliminates its profits [6]. Because the shipping price is highly dependent on head size, the harvest date of one-time mechanical harvesting is essential to determine the proportion of nonstandard-size broccoli and the total income for farmers. If the growth status of all broccolis in the field could be determined and predicted in the short term, it would be possible to set the optimal harvest date to reduce the number of nonstandard-size vegetables and minimize food losses.

**Table 1.** Meteorological data during the broccoli growth period.

Date	Mean temperature ( ^∘^C)	Mean precipitation (mm)	Sunshine duration (h)	Wind speed (m s^−1^)
2020.03	10.5	103.5	183.4	2.0
2020.04	12.8	228.5	218.1	1.9
2020.05	19.6	103.0	174.9	1.3
2021.03	12.5	143.0	186.5	2.0
2021.04	15.0	104.5	218.4	1.7
2021.05	19.7	72.0	145.0	1.3

Several studies have attempted to build models for predicting the optimal harvest date by taking temperature as an important factor into consideration. First, Marshall and Thompson [7,8] predicted the harvest date from the sowing date using solar radiation and temperature, without considering physiological or phenological development, and achieved an accuracy rate of ±7 days. Later, Tan et al. [9,10] built a similar model that included 2 growth phases from the emergence date (emergence to flower induction and from flower induction to harvest), and improved the accuracy to ±3.30 days. Grevsen [11] integrated a model based on temperature sum during the juvenile phase, head induction phase, and head growth stage and achieved a standard error of ±3 days. Lindemann-Zutz et al. [12,13] combined temperature with a stochastic variation of time to head induction; the model predicted the head size variation and reduced the times of necessary selective hand harvests (from 3 times to 1.8 ± 0.4 times) to achieve the same “harvest percentages not less than 80%” standard. However, the aforementioned models did not consider the differences among individuals, which is also a main cause of variation in head sizes at the time of harvesting. As Lindemann-Zutz et al. [13] reported, size variation in the heading stage causes an average of 79% variability in head size at the final harvest. Booij [14] also reported the mixed effects of individual size differences in the induction phase and temperature differences in the head growth phase on final head sizes. Since individuals in the same field often have quite similar solar and temperature conditions, considering the size differences among each individual would be valuable to further improve the accuracy of prediction models. However, manually measuring the size of all broccoli heads in the entire field using conventional methods is unrealistic.

Smart farming is expected to be a possible solution for measuring the sizes of all individuals. It involves new technologies such as remote sensing, high-throughput phenotyping, and artificial intelligence (AI) in agricultural production and has received considerable attention from researchers, farmers, and governments. The drone-based aerial pipeline provides a cost-efficient method to capture images for entire crop canopies. The field maps and three-dimensional (3D) canopy models can also be reconstructed by photogrammetry, which is also known as structure from motion and multiview stereo (SfM-MVS). Several studies extended this approach to estimate canopy architectural traits [15–17] and even traits for individual lettuce plants [18]. But such canopy- or individual-level trait phenotyping is not sufficient for the organ-level broccoli head size estimation. For example, in the Japanese market, the shippable broccoli head sizes are usually divided into 3 price levels (M: 11 to 12 cm, L: 12 to 13 cm, and 2L: 13 to 15 cm), whose sizes vary by a few centimeters. Therefore, centimeter- or even millimeter-level accuracy is fundamental to estimate size distribution and profit of whole field. To meet this accuracy demand, many organ-level applications often collect the images close to the ground (less than 1 m between a sensor and a plant) by a handheld camera or a tractor. Although several studies successfully proved the accuracy and feasibility of this close-range approach on small-area experimental fields [6,19,20], its efficiency is not always applicable to the large-area field with thousands of individuals. To apply the organ-level analysis to a large field using the drone-based approach, there are 3 challenges to be solved.

One challenge is the insufficient quality of the canopy model (2D field map and 3D point cloud) obtained from photogrammetry. Due to the plant structure movement in different drone images caused by wind, the canopy map and 3D model made by photogrammetry often have the effects of double mapping (ghost effect) and seamline distortion [21]. Many studies tried to fix the low quality using machine learning (ML) algorithms [22–24] or multispectral sensors [25,26]; these can be time-consuming and costly and are often not robust on different crops. The original drone images often have better quality but do not have pixel-level geographical coordinates; it is not possible to accurately match plants from images to corresponding locations in the field. One solution is to reuse the intermediate parameters during the 3D reconstruction. In the 3D reconstruction procedure, the relative rotation angle and position between the drone images and the object (field) have been calculated and calibrated by the ground control points (GCPs). Therefore, the transformation matrix from image pixel coordinates to real-world 3D geographical coordinates is available from the intermediate parameters. Duan et al. [27] and Guo et al. [28] tested this idea to find the real-world regions of interest (ROIs) on corresponding original drone images, while Lin et al. [21] developed their algorithms to fix the field map using the original drone images. But they did not publish any source code or tools. It is difficult and time-consuming to reproduce and use these research achievements directly.

The second challenge is the high labor cost of dealing with the complexity of image analysis. There are huge differences in sunlight, soil texture condition, and growing stage through the growing season. It is quite difficult to design a conventional computer vision (CV) algorithm to handle all types of variation at the same time. Deep learning (DL) has demonstrated considerable advantages in complex CV tasks [29]. According to whether the datasets require labeling or not, DL techniques can be categorized as supervised or unsupervised [30]. The supervised networks utilized in agricultural studies predominantly encompass convolutional neural networks (CNNs; ConvNet) [31], recurrent neural networks (RNNs) [32], and their variants. Conversely, the unsupervised networks encompass generative adversarial networks (GANs) [33], autoencoder (AE) [34], and other variants [29,30]. Although there are some agriculture studies that investigated the use of GAN for enriching training data [35], AE for managing unlabeled input data [36], and RNN for predicting time-series data [37], the majority of agricultural studies still focused on using CNN variants to extract features from input image data [29]. For instance, VggNet, GoogleNet, and ResNet are utilized for image classification. Similarly, DetectNet, Faster-RCNN, and YOLO are used for object detection. Additionally, Mask-RCNN, SegNet, and U-Net serve the purpose of semantic segmentation. For the studies concerning broccoli heads, Zhou et al. [38] proposed an improved ResNet for broccoli head grading (classification), García-Manso et al. [20] applied the Faster-RCNN for broccoli head detection and classification, and Blok et al. [39] applied the modified Mask-RCNN for broccoli head region segmentation. Although most of them took images under controlled conditions (indoor or inside a black box), these studies still need to label hundreds or thousands of training data. To fulfill the more complex outdoor tasks, a large amount of training data need to be manually labeled. Although there is a public training dataset for cauliflower available [40], it cannot be used directly on the broccoli head or another crop. Besides, labeling 14,000 individual plants manually as in that dataset is not feasible for building a new dataset for different crop or farmland applications.

To decrease the workload of training data annotation for DL solutions, transfer learning, data augmentation, and active learning are often used in plant phenotyping studies. For transfer learning, Desai et al. [41] transferred the ResNet-50 model that pretrained on the ImageNet dataset to better estimate the paddy rice heading date, while Blok et al. [6] transferred the Mask-RCNN model pretrained on the COCO dataset to better segment the broccoli heads. For the data augmentation, Zhou et al. [38] used geometric transformations (random cropping and rotation), and Blok et al. [6] applied more geometric transformations and added photometric transformations to them. A more advanced data augmentation strategy involves generating fake images [42]. All these studies have shown that data augmentation can improve model performance. For active learning, which iteratively trains the DL models and manually adjusts the outputs as new training data to further improve that model, Ghosal et al. [43] applied the active learning on sorghum detection and decreased the 75% annotation time and 85% annotation count. For agricultural applications, several aforementioned methods need to be used together to achieve better results. For one thing, it is due to the limited publicly available annotated agricultural data than CV datasets. As Blok et al. [6] reported, broccoli in the CV datasets are dishes on plates, rather than broccoli grown in the field. For another, many agricultural images demonstrate a higher level of occlusion and background clutter than traditional CV datasets, which leads to higher possibilities of mixing foreground and backgrounds [43]. To the best of our knowledge, there is no research on integrating all of the above solutions to maximize the reduction of data annotation workload currently.

The third challenge in high-throughput phenotyping is the computation costs involved. For instance, a high-resolution aerial image typically has a file resolution of over 5,000 by 3,000 pixels. Surveying and processing a 1-hectare field often require collecting over 200 images with over 4 trillion pixels. Handling such a massive amount of data directly in time using formidable computational power may not be costly for widespread applications. To address this high computation cost issue, the characteristic of agricultural tasks can be used to narrow down the regions for analysis. In agriculture, most crops or plants stay in the same position through the time-series data. Some difficult detection or segmentation tasks can be simplified when fusing with data collected from an earlier period. Mu et al. [44] obtained the convex hull of peach tree crowns from the easily analyzable winter time and used that to guide the difficult summer time crown segmentation task. Li et al. [45] located the maize positions on the seeding stage, and these positions were used to guide the difficult segmentation tasks when the maize canopy was closed with severe leaf overlapping. Similarly, for the broccoli tasks, the seedling stage was simpler than that during the flowering stage. The seedling position of the broccoli was almost the same as that of the broccoli head. Hence, the position detection results in the simple seedling stage could be a good reference for the subsequent complex flowering stage. This is expected to decrease the difficulty and workload of data analysis for broccoli head analysis. However, the feasibility of applying such a time-series data fusion approach to broccoli applications has not been reported to the best of our knowledge.

In this study, we tried to improve the temperature-based broccoli size prediction model using individual data measured by drone-based phenotyping. We overcame the aforementioned challenges and provided a highly accurate and labor-saving pipeline for broccoli head size estimation. Using this pipeline, we developed a prediction system for harvest dates to reduce on-farm food loss and improve the profits of farmers. The goals of this study were to (a) overcome the insufficient quality for organ-level analyzing from the drone-based photogrammetry, by using the open-source python package we published earlier; (b) decrease the labor cost for DL broccoli segmentation task; (c) decrease the computation costs for high-throughput time-series aerial data; (d) build the temperature-based growth model for individual broccoli and predict the head size in the following few days; and (e) conduct hypothetical harvesting and calculate the food loss and profit for the following days to determine the optimal harvest date. This pipeline also has great potential for seamlessly interfacing with other cabbage-like crops, including cauliflower, artichoke, and lettuce. Meanwhile, the use of a simple RGB sensor, not a complex integration of multiple or expensive sensors [i.e., multispectral and LiDAR (light detection and ranging)], makes it more applicable and user-friendly for the farmers, farming, and the economic sustainability of many economically and socially disadvantaged rural regions.

## Materials and Methods

**Fig. 1.**
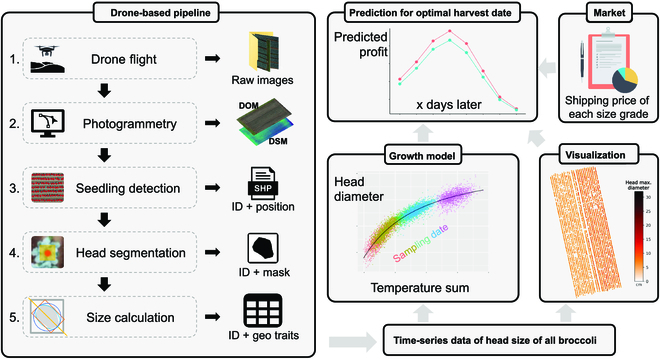
Workflow and schematic of the study method. First was the drone-based pipeline that was used to obtain the time-series head size information (geometry traits) of all broccoli heads during the growing season (the outputs of each step are the inputs of the next step). We then obtained a simple growth model between head size and temperature data, which were combined with price data obtained via a market survey to build the profit prediction model for the optimal harvest date.

The general workflow of the proposed pipeline is shown in Fig. 1 and the supplementary video (https://youtu.be/SYuOCVqgtrU). First, time-series data of all broccoli were collected and analyzed using a drone-based pipeline. To solve the challenge of insufficient quality for organ-level analysis, the regions of interest (ROIs) were backward projected on the original drone images that have better quality. To solve the challenge of labor-saving DL analysis, we integrated time-series data fusion, active learning, transfer learning, and data augmentation into the DL workflow by YOLO v5 (detection task) and BiSeNet v2 (segmentation task). The produced individual head sizes were used as the data source for the temperature-based growth model. Finally, a profit prediction model was generated according to the market price survey.

### Plot conditions and field data collection

**Fig. 2.**
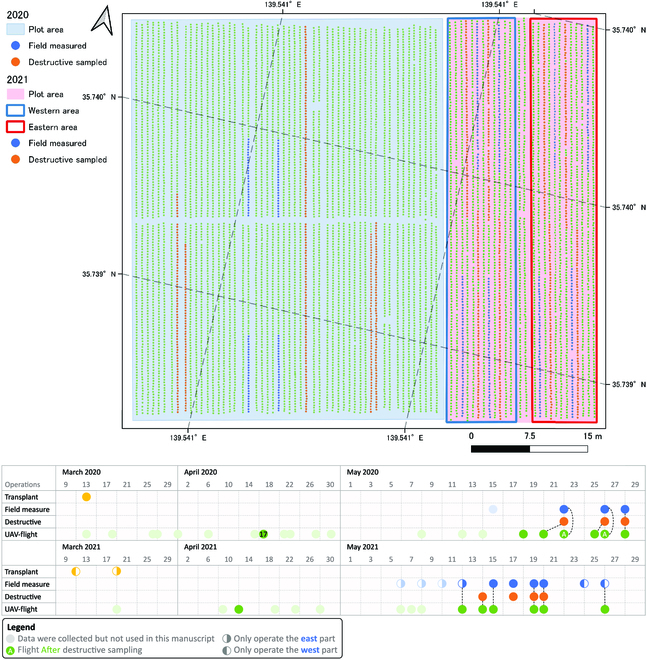
Plot conditions and timelines for field operation and data collection. The plots are connected but have not overlapped for 2 years. Field operations include transplanting, broccoli head nondestructive measurement in the field (field measure), broccoli head destructive measurement, and aerial survey. In the field map, the field measurements were conducted on the fixed blue positions on every occasion, whereas the orange positions were the final of all destructively sampled positions. In the timeline, the broken dashed line demonstrates how these data were paired.

Field trials were conducted at the experimental farm of the Institute for Sustainable Agro-ecosystem Services (ISAS), Nishi-Tokyo, Tokyo, Japan (35^∘^43′N, 139^∘^32′E) in 2020 and 2021 (Fig. 2). Detailed meteorological data during the growth period were collected by a local weather station and are shown in Table 1. The plot sizes were approximately 0.2 and 0.1 ha for 2020 and 2021, respectively. During the 2-year experiment, the same broccoli cultivar (Jet dome) was planted under the same field management. Machine planting of seedlings at 35-cm intervals in rows 70 cm apart is consistent with local commercial broccoli cultivation regimes.

The field data of the broccoli head size were manually measured as validation data (ground truth). This was conducted manually every 2 to 3 days using both destructive and nondestructive measurement methods. Nondestructive measures were conducted directly in the field, and destructive measures were conducted indoors. In 2020, the maximum broccoli head length [as head diameter (HD)] was measured by the visual judgment of the longest axis. A total of 360 (120 × 3 times) nondestructive and 434 destructive measurements of 7,438 individual broccoli were recorded.

In 2020, we identified the potential of the proposed algorithm. To further validate this algorithm, we improved the measurement method and increased the number of field samples in 2021. The maximum head length (as HD) was measured using the maximum value of the length in the 0^∘^, 45^∘^, 90^∘^, and 135^∘^ directions. To increase the variation in the broccoli head for each survey, there was an 8-day interval between seeding in the western and eastern parts (timeline in Fig. 2, yellow lunar phase). A total of 2,000 (400 × 5 times) nondestructive and 557 destructive measurements of 3,276 individual broccoli were recorded. To reduce the workload, only half of the area (east or west) was measured on a certain day (timeline in Fig. 2, blue lunar phase). HD measurements ranged from 2 to 25 cm.

### Drone-based pipeline

The pipeline included the following steps: (a) aerial data collection and data preprocessing (3D reconstruction by photogrammetry), (b) low labor-cost broccoli position detection using YOLO v5 at the seedling stage, (c) low labor-cost broccoli head segmentation using pretrained BiSeNet v2 on the original drone images, and (d) broccoli head size calculation.

#### Aerial data collection and preprocessing

After transplanting, several ground control points (GCP) boards were set at the 4 corners and within the field for aerial survery. This was an important resource for later 3D reconstruction and time-series alignment. In this study, all GCPs were measured using hemisphere real-time kinematic (RTK) differential Global Navigation Satellite System (GNSS) devices to obtain geographical coordinates. However, it is recommended but not mandatory. For developing regions without RTK services, the GCPs’ coordinates can be replaced by measuring distances (as scalebar corrector) among each GCP and building a referencing map at the very beginning. The relative coordinates of those GCPs on the referencing map can function the same as the actual geographic coordinates for time-series alignment.

Aerial images were collected using DJI (SZ DJI Technology Co. Ltd., China) Phantom 4 v2 (camera model FC6310s), DJI Mavic 2 Pro (camera model Hasselblad) in 2020, and DJI Phantom 4 RTK (camera model FC6310R) in 2021. The image resolution was the same at 5,472 × 3,648 pixels. The flight height in 2020 was initially 15 m and then decreased to 10 m when the broccoli head turned up. The flight height in 2021 was constantly 15 m. Most of the flights were conducted before the field operation, except on 2020 May 22 and 26. On both these days, the destructively sampled broccoli did not exist in the drone image; hence, the destructive data were linked to the previous flight (the black broken lines in the timeline in Fig. 2). For all other times, data collected on the same day were paired together.

The configuration of the computer for 3D reconstruction was as follows: Intel i9-7980XE CPU 2.6GHz, 64GB RAM, 2 NVIDIA GeForce GTX 1080Ti GPUs, and Windows 10 Pro 64-bit operating system. Pix4DMapper Pro (Pix4D, S.A., Prilly, Switzerland) was used to perform aerial photogrammetry on drone images. The default software parameters were used, and the digital orthomosaic map (DOM) and digital surface model (DSM) were produced. The open-source software package QGIS (www.qgis.org) was used to prepare shapefiles for field boundary and grid plots. The field boundary was a rectangular region that tightly wrapped around the broccoli farmland in the same direction as the ridge (Fig. 2). It was used to filter the noise outside the broccoli plot. It is split into several small grids with an edge length of around 2.5 m (approximately 1,000 to 1,500 pixels) and contained approximately 25 to 50 broccolis. The LabelMe annotation tool (https://github.com/wkentaro/labelme) was used to label the training data for the DL models. EasyIDP (https://github.com/UTokyo-FieldPhenomics-Lab/EasyIDP) [46] was used to locate and crop the same field region imagery on the original drone images (also known as backward projection or reverse calculation) when the DOM was not sufficiently clear for head segmentation.

#### Seedling position detection

**Fig. 3.**
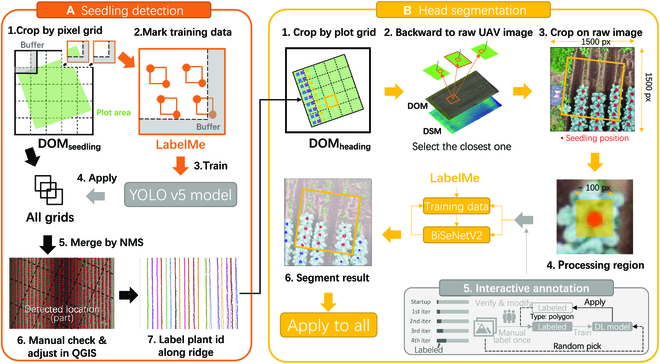
Workflow of broccoli (A) seedling detection and (B) head segmentation.

Here, we detected the broccoli positions on the simple seeding stage, and the general steps are introduced in Fig. 3A. The flight at approximately 1 month after transplanting was used to detect seedling positions (Fig. 2, dark green circle in April). In this period, most broccoli leaves were sufficiently large to be clearly observed from aerial images and the leaves did not overlap. The uniform light conditions and differentiation between leaves and backgrounds were also taken into consideration when selecting the most suitable time for detection task. Then, the full DOM was split into several small pixel grids (named “sectors,” Fig. 3A). The sector size was set to 1,300 × 1,300, which can maximize the efficiency of GPU memory usage for DL. For those broccoli heads located on the sector boundaries, they were split to 2 sectors and detected partly in both sectors. To avoid this issue, we buffered the sector with 200 pixels (slightly larger than the biggest broccoli head). The buffered area is illustrated by the gray L-shape in Fig. 3A on the lower right corner.

Subsequently, when this detection task was conducted in June 2020, the latest YOLO v5 (https://github.com/ultralytics/yolov5) detection model with default settings was trained and applied to all sectors. Due to the strong contrast between the broccoli seeding and soils, the detection model achieved a good performance with only 2 training sectors. During the writing of this manuscript, YOLO has made advancements to version 8, incorporating performance enhancements. However, for relatively simple head detection tasks with only 2 training images, where YOLO v5 has already achieved nearly perfect detection results (24 incorrect detections of 7,438 in 2020; 2 incorrect detections of 3,276 in 2021), there is limited room for significant improvements for other YOLO versions. Therefore, we have made the decision to maintain our workflow utilizing YOLO v5. The duplicated detection results in buffer zones were merged using the nonmaximum suppression (NMS) algorithm. The center point of the bounding box was then viewed as the broccoli position. Finally, we manually inspected and adjusted these detection results in QGIS, ensuring no missing or duplicate detection (Fig. 3A). The broccoli ID was given from the north to the south of each ridge and ridges from the west to the east by the ridge detection algorithm (Fig. 3A; please refer to the links in the Data Availability section for further details and the source code).

#### Broccoli head segmentation

The general workflow for broccoli head segmentation is illustrated in Fig. 3B. Only aerial surveys with clear broccoli heads were chosen for all 12 flight investigations over 2 years (Fig. 2, dark green circles in Mays). The field boundary (rectangle area containing broccoli) was divided into small grids (Fig. 3B1).

To improve the image quality for organ-level analysis, the same ROI on the original drone images rather than field map (DOM) were used for analysis by backward projection [46]. For each grid, its boundary and broccoli positions (detected in the previous section) were backward-projected onto the closest raw aerial image (Fig. 3B2). Surrounding the backward projection results, the original drone image was cropped into small sectors (1,500 × 1,500 pixel size, maximize the usage of GPU memory), with the grid boundary located in the center (yellow square in Fig. 3B3) and broccoli positions (red dots in Fig. 3B3).

To reduce the workload of DL data annotation and processing, the following techniques were used during this DL processing: (a) narrowed processing regions guided by time-series data fusion, (b) active learning (interactive annotation), and (c) transfer learning (pretrained model and data augmentation). The source code for this workflow is also available in the links in the Data Availability section.

For the first point, only the square area (approximately 100 × 100 pixels, slightly larger than broccoli heads) around the seedling positions was used for broccoli head segmentation (yellow square in Fig. 3B4). These narrowed processing regions not only decreased the processing area to save processing time on the unnecessary regions without broccoli heads but also eliminated the effects of soil and weeds in some contents.

For the second point, interactive annotation (also referred to as active learning-inspired annotation [43]) was applied to decrease the workload of label annotation (Fig. 3B5). Considering the promptness of the interactive annotation, BiSeNet v2 [47] was used as the segmentation model. BiSeNet v2 is a network structure that employs multiple branches to process inputs of different sizes to strike a balance between the efficiency and computational cost (Fig. 1 of [47]). The BiSeNetV2 network used in the present study was based on the https://github.com/CoinCheung/BiSeNet GitHub project. Initially, a small number of startup training data (approximately 5 to 10 broccoli heads per flight) were manually marked using LabelMe; then, the segmentation model was trained using the startup data. Next, images were randomly selected and applied to the segmentation results. These results were transformed into LabelMe JSON formats using Python scripts. Subsequently, manual adjustment produced annotations as new training data in LabelMe. The previous steps were iteratively repeated until no substantial adjustment was required for the newly applied results.

For the third point, the official pretrained model (https://github.com/CoinCheung/BiSeNet/releases/download/0.0.0/backbone_v2.pth) provided by BiSeNet was used for the transfer learning of broccoli head. Our data augmentation strategy used both geometric (G) and photometric (P) transformations, similar to [6]. The G strategy consisted of ShiftScaleRotate (shift limit = 0.5, scale limit = 0.2, rotate limit = 90) and VerticalFlip, which were used to solve the problem caused by our point-based or position-guided segmentation workflow. Ideally, the input images had only one broccoli in the middle of the input image, but in actual practice, the broccoli head position appeared randomly caused by bud-head growth shifting, or even with the probability of multiple or no broccoli heads in the input images. The P strategy simulated “cloudy, sunny” and “day, night” transitions using RGB shift (r shift limit = 25, g shift limit = 25, b shift limit = 25) and RandomBrightnessContrast (brightness limit = 0.3, contrast limit = 0.3) to address the effects of different weather and light conditions due to different flight investigations.

The verification dataset for the model performance evaluation was also prepared using the previous workflow. The modified intersection of union (IoU) was used as the evaluation metric. In this case, only the segmentation results inside the grid region (Fig. 3B, red polygon inside the yellow square) were chosen as the final results. The segmentation results attached to the grid bottom and right edge were also removed because of duplication with the neighboring grids. Here, we named this modified IoU inside the grid region as middle IoU, shortened to “Mid IoU”.

The segmentation model was first trained using only the 2020 dataset for several iterations until a good performance was achieved. The model was then applied to the 2021 dataset over several iterations. When the model performed well in both years, it was applied to all dataset images, and the segmentation results after the grid boundary filter were saved for the next procedure.

#### HD calculation

All the previous segmentation results (unit in pixels) were transformed back into the geographical coordinates for actual size (unit in centimeters), using the projective transformation function provided by scikit-image (https://scikit-image.org). For each polygon of the segmented broccoli head, the maximum side length of the minimum area bounding box was used as the HD.

To test the correlation between field-measured length (dependent variable) and aerial measured length (independent variable), the coefficient of determination (*r*^2^) using simple linear regression was used as the evaluator. To assess the trends in differences in broccoli size in detail, locally weighted scatterplot smoothing (LOWESS) regression and distribution comparison were also used.

### Optimal harvest time prediction pipeline

#### Prediction model for head size

The simple nonlinear regression model inspired by Grevsen [48] was used to predict the HD with time:

$$\ln\left(\mathrm{HD}\right)=a-b\cdot e^{-c\cdot T}$$

where *T* is the sum of the daily average temperatures, with a lower limit of 0 ^∘^C and an upper limit of 20 ^∘^C [9,49]. *a*, *b*, and *c* are the parameters to be determined.

**Fig. 4.**
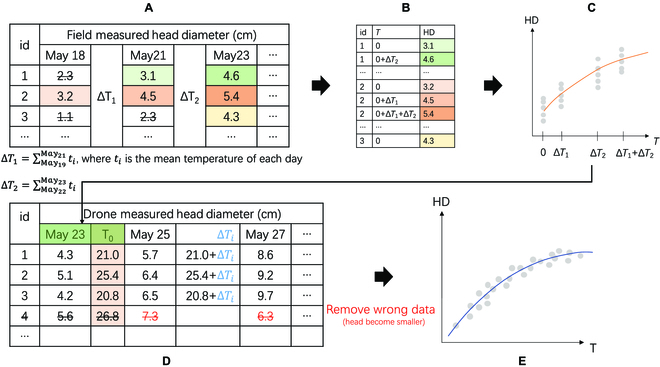
Data processing illustration for head size prediction model. All the numbers were just examples, not the actual results. (A) Field-measured diameter on different dates; the light color was used as the starting date with broccoli head size of approximately 3 to 3.5 cm. *T* is the sum of daily average temperature. Δ*T_i_* is the temperature sum deviation. (B) Reshaping of the previous table to a 2-column table for the regression analysis shown in (C). (D) The previous regressed model was used to initialize *T* from the HD. *T* on later days was added by the deviation Δ*T_i_*. (E) The previous data were used to regress the prediction model from *T* to HD.

First, the field-measured HD was used to build a model for drone initialization. The time-series nondestructive measured HDs (360 in 2020, and 2,000 in 2021) were selected. For each selected broccoli, based on a previous study [49], the starting date (i.e., the hypothetical date on which the broccoli head became manually detectable) was set, and its HD was approximately 3 to 3.5 cm. The sum of the daily average temperatures (*T*) was normalized based on the starting date as 0 (Fig. 4A and B). The model parameters were then regressed and calculated (Fig. 4C).

The drone-based measurements also started when the HD was approximately 3 to 3.5 cm. To obtain the proper normalized *T*_0_ for the first flight (HD around 9.5 cm), the previous model was inverted to calculate *T*_0_ from the HD (Fig. 4D). Then, the sum of the daily temperature (*T_i_*) of later days (*i*) was calculated (Fig. 4D). That model was then regressed and calculated in a similar manner as the prediction model (Fig. 4E). Based on this model, we calculated the head size using the time-variable *T* for all flights after the first aerial survey.

To verify the accuracy of the prediction, the model generated for one of the study years was used to predict the head size for the other year. The results were compared between the predicted head size and that estimated using drone-based measurements. The *r*^2^ obtained using simple linear regression was used as the evaluator.

#### Market price survey

In the Japanese market, broccoli head sizes are usually divided into 3 levels (M: 11 to 12 cm, L: 12 to 13 cm, and 2L: 13 to 15 cm), with L usually having the highest shipping price. To set the shipping price for each grade, we interviewed several local officers of the Japan Agricultural Cooperatives (i.e., the largest cooperative organization in Japan). Because the price of each grade varies depending on the season and location, we used 2 types of shipping prices for the experiment: one where the price difference between each grade was the largest (case 1), and one where the difference was the smallest (case 2).

#### Income estimation for hypothetical harvesting

It was assumed that all individuals were harvested at the same time each day from 2020 May 18 to 28 and 2021 May 12 to 26. The drone-predicted HD was used to calculate income (from the first day of the aerial survey to 10 days later in 2020 and 14 days later in 2021). The number of individuals of each size standard (M, L, and 2L) was counted for each date. The shipping price of size L was assumed to be $1.00, and the prices of other sizes were calculated from market survey interviews. Nonstandard-size broccoli (<11 cm and >14 cm) could not be sold, and the income was set to zero. Finally, the total income on each harvest date was calculated by multiplying the number and shipping price of each size grade (for both cases with a shipping price difference). The date with the highest income was selected as the optimal harvest date.

## Results

### Drone-based pipeline

#### Broccoli position detection

**Fig. 5.**
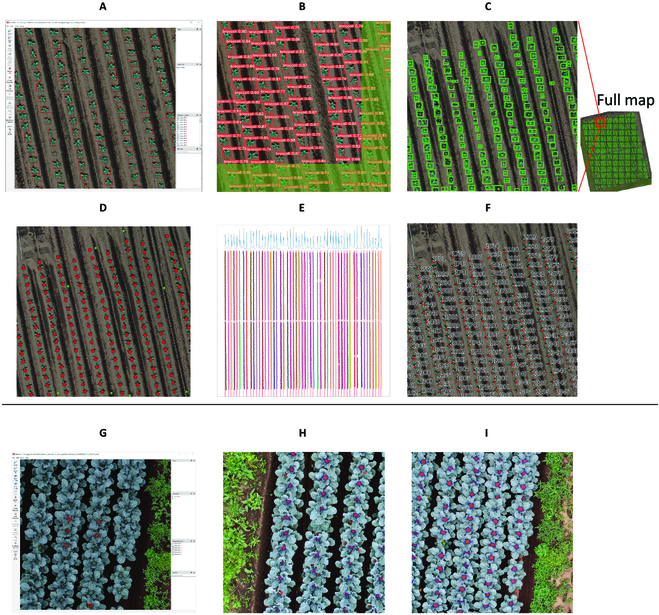
Examples of 2020 broccoli seedling position detection (A to F) and head segmentation by interactive annotation (G to I). (A) One annotated training data by LabelMe. (B) YOLO v5 detected results; the green part is the buffer zone to avoid the broken broccoli at the edge. (C) Duplicate detection in buffer zone removed by the nonmaximum suppression (NMS) algorithm. Black shows removed duplicate detection, green shows those that were retained, and green dots are the center points as the broccoli position. (D) Red dots show the manually adjusted positions by QGIS. (E) Ridge detection by identifying the peak of point distribution. (F) Automatic placing of plant ID along the ridge. (G) Startup training data annotation made by LabelMe; only a few annotations were required. (H) After the first iteration. The red polygons are the segmentation results (as auxiliary annotations) trained by the startup data, and the blue polygons are manually adjusted according to the previous results. (I) After the fourth iteration, almost no manual adjustment was required in this case.

To provide a general understanding of this procedure, Fig. 5A to F shows some intermediate results during broccoli position detection. For training data preparation, considering the clear differences between the broccoli plant and brown soil, only 2 representative sectors were chosen as training images. All broccoli were labeled using bounding box (rectangle) annotation in LabelMe (Fig. 5A, example sector). The detection model performed as expected in the other sectors (Fig. 5B: one example sector). The green mask in Fig. 5B shows the buffer zone that overlapped with neighboring sectors, with the aim of avoiding incomplete broccoli detection on the sector boundary. When merging the detection results for all sectors, duplicate detections were removed by the NMS algorithm (black rectangles in Fig. 5C). When adjusting the zoom to the full map view, the removed black duplicates were clearly distributed on the sector boundaries (grid lines). In Fig. 5D, the green dots show the misdetection results, and the red dots indicate the final positions used by manual adjustment. Figure 5E shows the results of ridge-line detection, and Fig. 5F shows some results of the automatic broccoli ID assignment. In general, broccoli position detection functioned as expected with only labeling 2 sectors as training data in a few minutes; afterward, around a half-hour manual postprocessing for fixing the misdetection and position devitation is required.

#### Broccoli head segmentation

To demonstrate the interactive annotation procedure, Fig. 5G to I and Table 2 provide example images and a simple summary from the first to the last iteration. As startup training data, one image was randomly selected from each aerial survey in 2020 (6 in total), and only around 5 broccoli heads were annotated as simply as possible (Fig. 5G). The BiSeNet model (v0) was then trained using this annotation. Afterward, the v0 model was applied on a few randomly selected images for each aerial survey (Fig. 5H, red mask). These results were manually adjusted and saved as new training data of v1 model (Fig. 5H, blue boundary). The previous steps were iteratively repeated until the model achieved good segmentation results. A total of 30 validation sectors were also randomly selected, and the v2 model results with manual adjustment were applied similarly to get the validation data with low labor costs.

**Table 2.** Middle intersection of union (Mid IoU) changes around each iteration in weakly supervised learning. Four iterations were first conducted on the 2020 dataset only and then applied to the 2021 dataset.

Training data file number	Model version	Training time (s)	Mid IoU in 2020 (%)							Mid IoU in 2021 (%)						
			May 18	May 20	May 22	May 25	May 26	May 28	Mean	May 12	May 14	May 15	May 19	May 20	May 26	Mean
Startup 2020x6	v0	100.8	69.82	82.08	84.49	77.82	68.09	86.60	78.15	-	-	-	-	-	-	-
v0 add 2020x6	v1	166.1	76.28	87.60	88.60	84.16	81.75	89.02	84.57	-	-	-	-	-	-	-
v1 add 2020x9	v2	420.4	84.44	89.35	89.07	85.94	88.17	89.76	87.79	-	-	-	-	-	-	-
v2 add 2020x14	v3	649.2	84.91	89.77	89.30	86.56	90.12	90.03	88.45	-	-	-	-	-	-	-
v3 add 2020x12	v4	1,085.2	83.37	90.04	89.57	86.49	90.15	90.35	88.33	47.09	79.98	80.29	91.77	91.26	84.56	79.16
v4 add 2021x6	v5	1,267.5	85.04	90.20	90.35	86.13	90.63	90.49	88.81	85.07	88.84	91.24	94.59	94.55	95.88	91.70

The detailed model performance for each iteration version is presented in Table 2. With our proposed labor-saving strageies for DL, even a startup with only 30 head annotations achieved a Mid IoU of 78.15%. The model performance improved considerably after 4 iterations and achieved a Mid IoU of 88.33% after the 4th iteration (Fig. 5I). Then, when the v4 model trained by 2020 data was applied to the 2021 data, the performance decreased to 79.16%. It is reasonable because we just quickly trained an “overfitting” segmentation model with a few training data from 2020. The model performance improved to 91.70% after one additional iteration with 6 training data points added from 2021. Meanwhile, broccoli head segmentation was more challenging at an early stage (2020 May 18 and 2021 May 12, with the lowest Mid IoU) when the head size was small. In general, we obtained an “overfitting” broccoli segmentation model by only labeling 53 sectors (images), and 47 of them were manually adjusting model-produced segmentation results. Compared to other DL segmentation studies, Zhou et al. [38] trained the broccoli head segmentation and grading model (IoU = 84.8%) with 506 images under a controlled greenhouse environment, Tassis et al. [50] trained the segmentation models (UNet IoU = 94.25% and PSPNet IoU = 93.54%) for diseases and pests of coffee leaves with 800 images, and Osorio et al. [51] trained the weed segmentation model (accuracy = 89.0%) with 913 samples. Our proposed labor-saving approach achieved a similar model performance with only 20% workload in data annotation.

#### HD calculation and validation

**Fig. 6.**
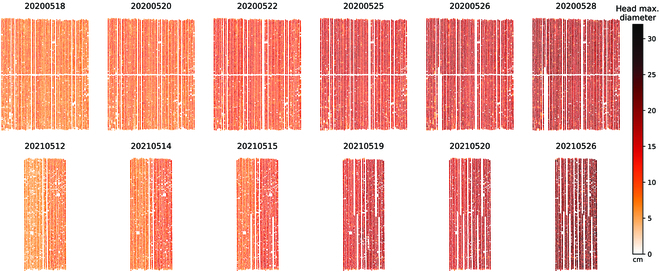
Maximum diameter of all broccoli heads by drone-based phenotyping.

The individual broccoli HDs during the growing season were successfully calculated through our proposed drone-based phenotyping workflow. The full map of all broccoli HDs is shown in Fig. 6. The gradually missing parts in the picture are due to destructive sampling. In 2020, all broccolis were transplanted at the same time (March 13, Fig. 2), resulting in a synchronized growth pattern. However, in 2021, the transplanting time for broccolis in the eastern and western areas differed by 8 days (March 11th for the western area and March 19th for the eastern area, as shown in Fig. 2), leading to noticeable differences in growth rates (2021 May 12 to 15 in Fig. 6). Nevertheless, since the growth rate of broccoli plants slows down as they reach a certain size, toward the end of the growth period (as indicated by the last 2 columns in Fig. 6), most broccoli plants tend to have similar sizes. At the same time, individual size differences mentioned by Lindemann-Zutz et al. [13] and Booij [14] can also be observed in Fig. 6, namely, those points with sudden changes in local color. When not taking the individual sizes into consideration, based on the general size colors in Fig. 6 and the shipping sizes for the Japanese market (11 to 15 cm), the optimal harvest dates should be between 2020 May 22 to 25 and 2021 May 15 to 19.

**Fig. 7.**
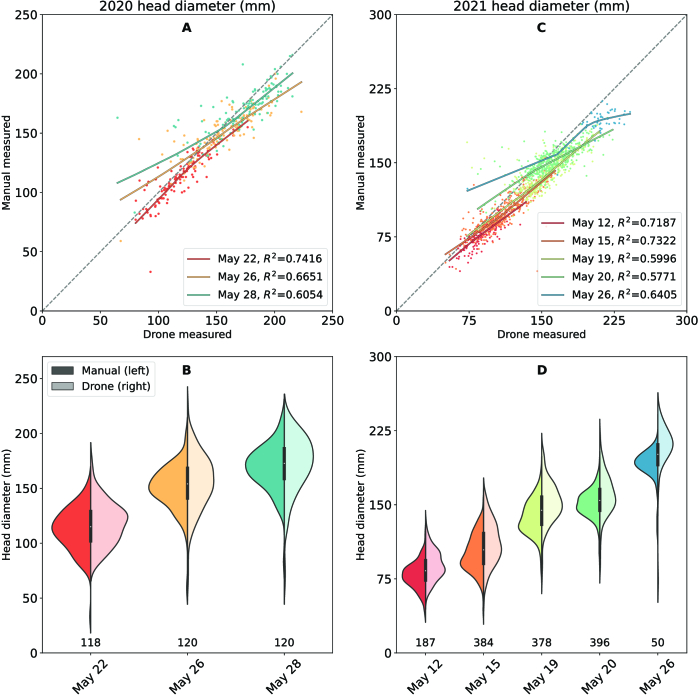
Comparison of broccoli HD measured from drone images and obtained using manual field measurements. Different colors represent different investigation dates. In (A) and (C), the curved solid lines represent the trends by locally weighted scatterplot smoothing (LOWESS) regression. In (B) and (D), the violin plots show comparisons of the value distribution; darker colors (left part) show manual field measurements, brighter colors (right part) show drone measurements, and the values below show the broccoli count.

To validate the accuracy of these drone-measured diameters, we compared the results with field-measured diameters. We found a good correlation (*R*^2^ ≥ 0.57) between the maximum diameters of the drone-measured broccoli head and those measured manually in the field (Fig. 7). The aerial survey tended to underestimate broccoli growth (trend line above the reference line). It is reasonable due to the leaf occlusion. However, the overall distribution of the estimated head size was almost the same between the 2 groups (drone versus manual, Fig. 7C and D). It can be explained that, overall, the methods of drones can accurately represent the broccoli HD distribution of the entire field. We also calculated the root mean square errors (RMSEs), which were 9.730, 11.02, and 12.51 mm for 2020, and 7.01, 9.58, 11.06, 9.15, and 8.97 mm for 2021. This error is acceptable, since Blok et al. [39] reported a total RMSE = 9.7 mm on their close-range approach, which mounted the camera on the tractor frame with only around 1 m between the sensor to the broccoli head. Our aerial survey at 15 m flight height (flight speed 5 m∙s^−1^, 30 min for the entire field with 3,000+ broccolis) has much higher survey efficiency than the ground survey (tractor speed 0.14 m∙s^−1^, around 16 min with 2 rows and 122 broccolis) made by Blok et al. [39]. Both approaches obtained similar accuracies with the field measurements.

### Optimal harvest time simulation

#### Prediction model for head size and swap validation

**Fig. 8.**
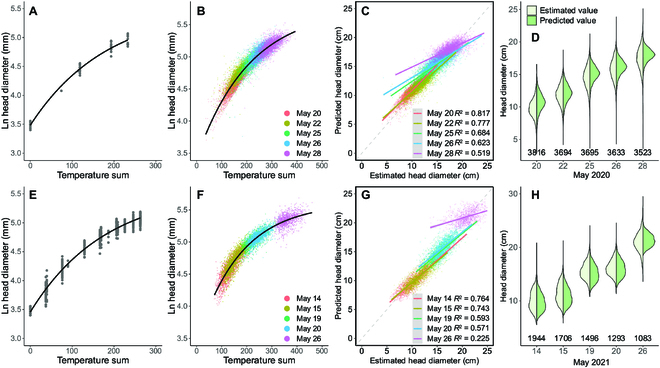
HD prediction models and validation for 2020 and 2021. (A and E) Initialization models for drone data regressed by the field-measured data for 2020 and 2021, respectively. (B and F) Final prediction models by the drone-measured data for 2020 and 2021, respectively. (C) Comparison between the 2020 drone-measured HD and the HD predicted by the 2021 model (swapping validation). (G) Comparison between the 2021 drone-measured HD and the HD predicted by the 2020 prediction model. (D and H) Distribution of the previous comparison.

The initialized models using field measurements in 2020 and 2021 are shown in Fig. 8A and E, respectively. The parameters were *a* = 5.53, *b* = 2.05, and *c* = 0.00546 for 2020, and *a* = 5.57, *b* = 2.11, and *c* = 0.00558 for 2021. These initialized models were used to calculate *T*_0_ of the first flight (Fig. 4D). *T* values on other dates were calculated using meteorological data. Comparing those parameters and the trends in Fig. 8A and E in 2 years, the initialized models for the same broccoli and field treatment did not have significant differences. However, this does not mean that the model can be directly applied to other varieties of broccoli and farmland without modification. We did not establish a general growth model for all broccoli varieties. On the contrary, we prefer customizing unique initialized models for each farmland with low labor costs. This has more practical significance compared to a general growth model.

After getting the initialized models by field measurements, all the drone-measured HDs (date as *x*, HD as *y*) were converted to temperature-based model format (temperate sum as *x*, Ln(HD) as *y*), by using the meteorological data (daily temperature delta, Δ*T_i_*) and the initialized model (calculate the starter *T*_0_), as shown in Fig. 4D. The converted results for all aerial survey dates in 2020 and 2021 were those scatter points in Fig. 8B and F, respectively. The regression models (ln(HD) = *a* − *b* · *e*^−*c* · *T*^) for these converted results were then generated and used as the broccoli HD prediction models (Fig. 8B for 2020; Fig. 8F for 2021). The parameters were *a* = 5.75, *b* = 2.33, and *c* = 0.00475 for 2020 and *a* = 5.58, *b* = 1.94, and *c* = 0.00616 for 2021. Although the difference between the 2 years may not seem significant from the parameters, when reflected in the trend of regression models (Fig. 8B and F) after accumulated temperature conversion, it can be noticed that the growth curves of the 2 years are not the same. According to the meteorological data (Table 1), although the Mays in 2020 and 2021 have close mean temperatures (19.6 and 19.7 ^∘^C), the year 2021 has 29 h of sunshine duration and 30 mm of mean precipitation shorter than those in 2020. When these factors are applied to the growth of broccoli, the climate variations in 2 years result in different growth curves in the model. That is why we encourage not to rely on general models and instead establish independent models for each year.

Although it is ideal to establish an independent model based on each year as described above, in cases where there is a lack of data for the current year, the model from the previous year can also be used to obtain approximate results. To validate this point, the initializing model and the prediction model for 1 year (e.g., 2020 models in Fig. 8A and B) were used to predict the broccoli HD in the other year (e.g., 2021). The cross-year predicted values were compared by the actual drone-measured HD (Fig. 8C and G). The predicted sizes and drone measured were highly correlated with the acceptable *r*^2^ > 0.57 at the early stage (2020 May 20 to 26; 2021 May 14 to 20), while the last day (late stage) showed bad correlations (*r*^2^ = 0.519 in 2020; *r*^2^ = 0.225 in 2021). However, according to the size distribution maps (Fig. 6), the broccoli sizes at the late stage were far beyond the market standard. In actual production activities, it will not happen. Therefore, the poor results in later predictions will not have a considerable impact on guiding the actual production. At the same time, it can be found that although there is a large gap in one-on-one comparison (Fig. 8C and G), the overall distribution is still relatively close (Fig. 8D and H). This indicated that previously collected data can be used to predict growth before the harvesting period.

#### Income estimation and optimal harvest date

**Fig. 9.**
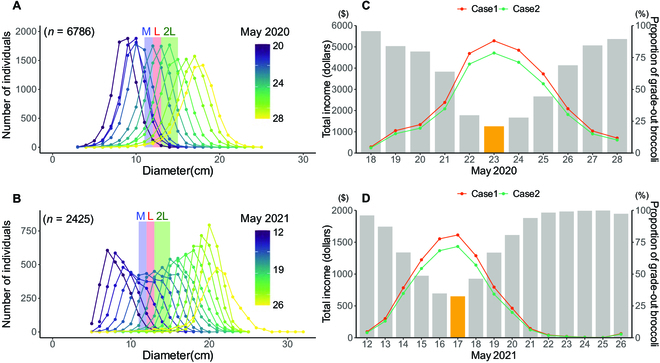
Distribution of predicted HD in (A) 2020 and (B) 2021 trials. The M, L, and 2L sizes meet the shipping standard in the Japanese market (M: 11 to 12 cm, L: 12 to 13 cm, and 2L: 13 to 15 cm). The proportion of nonstandard-size broccoli and total income assuming that all individuals were harvested for each date in the (C) 2020 and (D) 2021 trials. Yellow pillars indicated the optimal harvest date generating the highest income and lowest wasted broccoli. Case 1 is the largest price difference between each grade (indicates highest profit), and case 2 is the smallest difference (indicates lowest profit).

Based on the initialization and prediction models, we calculated the size distribution of all broccoli during the harvest period (Fig. 9A and B). The proportion of nonstandard-size broccoli and the total income for each date were then calculated (Fig. 9C and D). In the 2020 trial, May 23 was the optimal harvest date, i.e., the date on which the proportion of nonstandard-size broccoli was minimized and total income was maximized. In the 2021 trial, May 17 was determined as the optimal harvest date. In both cases, we found that a 1-d shift in harvest from the optimal date could lead to considerable income loss (3.7% to 20.4% reduction). The price differences among size grades did not affect the estimation of the optimal harvest date (cases 1 and 2 in Fig. 9C and D).

## Discussion

Reducing on-farm food loss (e.g., nonstandard-size vegetables) is one of the prominent goals of sustainable development in agricultural production. The main aim of this work is to test the use of drone-based digital measurements of broccoli head size, their usage in monitoring broccoli growth, and prediction of optimal harvest time in terms of economic returns for different sellable size classes. In this study, we created a harvest date prediction system based on aerial survey, ML/DL, and a growth model by predicting the short-term change in the head size of all individuals (>3,000) in the entire broccoli field. Our experiments demonstrated that (a) the head sizes estimated by aerial survey were highly correlated with the field measurements, (b) the proportion of nonstandard-size and the total income calculated by the hypothetical harvesting changed dramatically between harvest days, (c) predictions for the few days following a particular date of aerial survey were highly correlated with those estimated by aerial survey taken on that date, and (d) the optimal harvest date (i.e., the date for the minimum proportion of nonstandard-size broccoli and maximum income) could be predicted with high accuracy. These results suggested that our prediction system for the optimal harvest date of broccoli will benefit farmers by reducing food loss and increasing their income. Although the current study focused on broccoli as a model system, this framework could be readily applied to other similar vegetables such as cauliflower, artichoke, and cabbage. Thus, our case study shows that smart farming techniques have great potential to contribute to the sustainable development of vegetable production.

This study showed that the proportion of nonstandard-size broccoli and the total income changed rapidly depending on the harvest date. For example, 1 day later or earlier than the optimal harvest date increased the number of nonstandard-size broccoli by approximately 5% and decreased the total income by approximately 20%, and 2 days later than the optimal harvest date increased the number of nonstandard-size broccoli by approximately 15% and decreased the total profit by approximately 40%. To the best of our knowledge, such temporal changes in the number of nonstandard-size vegetables and the total profit for different harvest dates have not previously been calculated because no technique was available to measure thousands of individual vegetables multiple times with high accuracy. Interestingly, the optimal harvest date was largely unaffected by differences in the shipping price for each grade (Fig. 9C and D). The optimal harvest date was determined by the spatial variation in broccoli growth, regardless of the shipping price of each grade. The difficulty in setting the harvest date for mechanical harvesting owing to large spatial variation is a common issue in broccoli and other vegetable farms. Thus, predicting the optimal harvest date using our system (or similar systems) has the potential to reduce on-farm food loss and increase the income of vegetable farmers worldwide.

In addition to estimating temporal variations in head size distribution, our pipeline could visualize spatial variations in individual head size (Fig. 6). In the 2021 trial, spatial variation in broccoli size was intentionally created by planting seedlings on 2 different dates in the eastern and western areas of the field (8-day intervals). When we visualized the individual head sizes, the difference between the eastern and western fields was evident. However, it was difficult to visually observe the differences on the ground. For example, the difference in average head size between the east and west on 2021 March 12 was only 3 cm. Although the uneven growth between the west and east fields in this trial was intentional, such spatial variation can occur unintentionally and is a major challenge, especially in large-scale agriculture [52]. In such a large-scale uneven field, farmers can divide their field into several areas and harvest broccoli multiple times. Using our framework, farmers may be able to visualize the spatial variation of their fields, predict their short-term growth, and determine the optimal spatial and temporal harvest strategy.

Although the pipeline we developed highlights the benefits and importance of aerial survey powered by ML/DL for sustainable agricultural development, there are some limitations to its use. First, our system is neither fully automated nor app-based; therefore, farmers without computer science backgrounds cannot use this system directly in their own fields. However, because the source code is open to the public (https://github.com/UTokyo-FieldPhenomics-Lab/UAVbroccoli), local agricultural institutes and agricultural companies are able to modify and use the system according to their target. This study is definitely not a one-stop solution, but is a pioneer in real agriculture applications. Second, unlike traditional manual methods with limited throughput, the proposed method can sample every plant in the field at much higher frequency, leading to higher temporal and spatial resolution. The large amount of data generated by this method is therefore appropriate for data-driven modeling, which could lead to breakthroughs in smart farming. Third, manual inspection of the seedling position detection is required; this step cannot be omitted because this result is the basis for subsequent broccoli segmentation. Detection omissions, duplications, and drifts need to be checked manually on a case-by-case basis. Although it saves considerable effort compared to adding them manually one by one, this inspection still requires several hours to complete in large-scale fields. Additionally, if a broccoli plant dies before the flowering stage, there is a risk of wrong head segmentation generating incorrect results (but in some cases that we observed, it gave an empty segmentation result and was easily discarded). Fourth, the problem of leaf occlusion has not been solved, which remains a challenging problem for plant phenotyping [29]. As broccoli heads are essentially round, one approach is to restore the roundness of the stubs. The circularity and eccentricity of the broccoli head may be used to describe the severity of occlusion. The least squares for round fitting can be used, or the DL framework “occlusion-aware region-based convolutional neural network” (ORCNN) can be applied to obtain improved recovery results [39]. However, it requires a depth camera and image pairs before and after occlusion as training data are collected on the ground, which is inconvenient for current aerial survey but warrants further study. For example, multispectral and even LiDAR sensors are becoming increasingly cheap, combined with the rapid development of AI algorithms, suggesting that this problem could be resolved without unaffordable cost increases. Also, integration of the method with other common management practices such as mulching films with bioplastics could assist in the identification of plants and broccoli heads, particularly when used in conjunction with a multispectral sensor. Finally, hardware and software instrument costs should not be omitted. The drone with RTK ($6,500), 3D reconstruction software ($3,499), and a high-performance computer ($6,000) for computation used in this chapter would limit the pipeline’s widespread use. However, even for a small farm (0.2 ha) with 7,000 broccoli plants, only 2 days difference from the optimal harvest date can result in an income loss of almost $2,000. The feasibility of our pipeline on larger farms is also worth being tested in the future. For companies that provide this type of agricultural consulting service, this one-off investment can be offset by the increased profit of many producers. For those economically and socially disadvantaged rural regions, the RTK or the expense of a base station should not be mandatory. It can be replaced by setting more GCP boards and measuring distances among them as scalebar correctors, to get similar results with relative geographical coordinates. It was suggested that cooperating with local broccoli farmers to test the proposed system without RTK dependences and keeping improving the algorithm performance on the occlusion area will be needed in futher work. The head quality and transport costs were also suggested to be integrated into the system to refine its applicability.

## Conclusion

This is a demonstrable application of aerial phenotyping technology to assist farmers in optimizing financial returns and minimizing food waste rather than the majority of digital agriculture studies that are aspirations and lack the pipeline to actually help farmers in an applied context. In this study, using aerial survey and ML/DL, we developed a system for estimating and predicting the head size of whole broccoli with high accuracy and showed that the system can predict the optimal harvest date. This drone-based prediction system is based on several technical improvements and requires minimal labor and computational costs. Therefore, it could be applied to support broccoli farming and, with modifications, to a variety of similar vegetables (i.e., cabbage, cauliflower, artichoke, and lettuce). Because our developed pipeline uses a simple sensor, not a complex integration of multiple sensors, it would be more applicable and user-friendly for economically and socially disadvantaged rural regions, and it has the potential to be widely adopted by vegetable farmers worldwide.

## Data Availability

The source code used in this manuscript can be accessed at https://github.com/UTokyo-FieldPhenomics-Lab/UAVbroccoli. All original drone image data can be accessed by Google Drive upon request (224 GB for 2020 and 72 GB for 2021). Material: An illustration video (https://youtu.be/SYuOCVqgtrU) about the general pipeline of this study. The background music used in this video is copyright-free music from freepd.com.
